# Repurposed agents in the Alzheimer’s disease drug development pipeline

**DOI:** 10.1186/s13195-020-00662-x

**Published:** 2020-08-17

**Authors:** Justin Bauzon, Garam Lee, Jeffrey Cummings

**Affiliations:** 1 School of Medicine, University of Nevada, Las Vegas (UNLV), Las Vegas, NV 89154 USA; 2 Cleveland Clinic Lou Ruvo Center for Brain Health, Las Vegas, NV 89106 USA; 3 Chambers-Grundy Center for Transformative Neuroscience, Department of Brain Health, School of Integrated Health Sciences, University of Nevada, Las Vegas (UNLV), Las Vegas, NV 89154 USA

**Keywords:** Alzheimer’s disease, Clinical trials, Repurposing, Dose, Formulation, Disease-modification

## Abstract

**Background:**

Treatments are needed to address the growing prevalence of Alzheimer’s disease (AD). Clinical trials have failed to produce any AD drugs for Food and Drug Administration (FDA) approval since 2003, and the pharmaceutical development process is both time-consuming and costly. Drug repurposing provides an opportunity to accelerate this process by investigating the AD-related effects of agents approved for other indications. These drugs have known safety profiles, pharmacokinetic characterization, formulations, doses, and manufacturing processes.

**Methods:**

We assessed repurposed AD therapies represented in Phase I, Phase II, and Phase III of the current AD pipeline as registered on ClinicalTrials.gov as of February 27, 2020.

**Results:**

We identified 53 clinical trials involving 58 FDA-approved agents. Seventy-eight percent of the agents in trials had putative disease-modifying mechanisms of action. Of the repurposed drugs in the pipeline 20% are hematologic-oncologic agents, 18% are drugs derived from cardiovascular indications, 14% are agents with psychiatric uses, 12% are drug used to treat diabetes, 10% are neurologic agents, and the remaining 26% of drugs fall under other conditions. Intellectual property strategies utilized in these programs included using the same drug but altering doses, routes of administration, or formulations. Most repurposing trials were supported by Academic Medical Centers and were not funded through the biopharmaceutical industry. We compared our results to a European trial registry and found results similar to those derived from ClinicalTrials.gov.

**Conclusions:**

Drug repurposing is a common approach to AD drug development and represents 39% of trials in the current AD pipeline. Therapies from many disease areas provide agents potentially useful in AD. Most of the repurposed agents are generic and a variety of intellectual property strategies have been adopted to enhance their economic value.

## Background

Alzheimer’s disease (AD) is a progressive neurodegenerative disorder with a rising prevalence due to the global increase in the aging population [1]. It is estimated that one in 85 people will be living with an AD diagnosis by 2050 [2]. An urgent need exists to identify effective interventions that will combat this disabling and ultimately fatal disease. Attempts to find new therapies for AD have been made by academic centers and biopharmaceutical companies: identifying new chemical entities, optimizing them for human use, and investigating their effects in clinical trials. However, the lack of an approved drug for AD by the Food and Drug Administration (FDA) since 2003 demonstrates the complexity of this objective [3].

Efforts to produce new treatments for AD are focused primarily on identifying new compounds that may improve symptoms, slow disease progression, delay the onset, or ultimately prevent the disease. An alternative approach to advancing new treatments is the development of drugs approved for a non-AD-related indication that may impact the biology of AD through on-target or off-target mechanisms. This repurposing strategy has succeeded in other conditions and has promise for AD since many FDA-approved agents exhibit AD-relevant effects evident through in vitro, animal, epidemiologic, and observational studies [4–9].

To better understand repurposing in the AD drug development process, we conducted an analysis of all ongoing AD clinical trials involving FDA-approved drugs. We analyzed trial data reported on ClinicalTrials.gov, a comprehensive US government database with mandated registration of all clinical trials conducted within the US. We examined all currently ongoing trials that involved repurposed agents and compared this to all drugs in the AD pipeline [10–14]. We also compared ongoing trial activity of repurposed AD drugs as reported on the European Union Clinical Trials Register (EUCTR). Our goal was to examine how repurposing efforts in the AD drug pipeline compares to overall drug development activity in the United States (US) and international pipelines and to provide insight into repurposing as a drug development strategy.

## Methods

This study is based on the activity of clinical trials reported on ClinicalTrials.gov. By US law, all clinical trials conducted within the US must be registered within 21 days of the enrollment of the first participant [15, 16]. ClinicalTrials.gov is a comprehensive and valid data source for the study of clinical trials conducted in the US. Most, but not all, non-US trials are registered on clinicaltrials.gov; Phase I trials conducted outside the US are often not registered and our findings may underrepresent the agents populating global Phase I efforts.

We used a previously constructed trial database that included the data of interest [10–14] and report on all trials currently testing repurposed agents in Phases I, II, and III. The results reported here are based on trials registered on ClinicalTrials.gov as of February 27, 2020. We used ClinicalTrials.gov labeling and included trials that were recruiting, active but not recruiting, enrolling by invitation, and not yet recruiting. We do not include trials of stem cell therapies among the interventions reviewed.

Our focus on repurposed agents places an importance on drugs with an FDA-approved indication(s). Agents were considered to be repurposed based on the information recorded on Drugs@FDA (fda.gov/drugsatfda), a US government database containing drug products approved since 1939 that is updated daily [17]. The approved use for each drug was determined by the most recent label provided by Drugs@FDA. Some drugs have more than one FDA-approved indication, and this is noted.

Agents were further classified based on the therapeutic area for which each drug is prescribed as determined by its approved indication. Based on this classification, we examined the therapeutic area origin of agents being studied across all AD trials. Some agents are being studied in multiple ongoing trials; we defined these agents as “unique” agents. Varieties of a particular compound seen in two or more trials (i.e., dosing, routes of administration) were noted.

Some trials are investigating multiple repurposed drugs in separate treatment arms (e.g., telmisartan vs perindopril) or in combination (e.g., losartan, amlodipine, and atorvastatin as a single treatment). For the purposes of this study, we treat the former as separate therapeutic trial agents; the latter is considered a single agent with respect to its clinical trial because the intent of the trial is to study the effects of the fixed combination as a single therapy.

A drug’s putative mechanism of action (MOA) in AD was determined from the information on ClinicalTrials.gov or from a comprehensive review of the literature for the specific agent. We grouped the mechanisms into disease-modifying therapies (DMTs) or symptomatic agents. The latter is further divided into cognitive-enhancers or those that address neuropsychiatric and behavioral symptoms. We identified an agent as a DMT when the purpose of the intervention was to modify the underlying disease biology and to delay the onset or slow the progression of the disease. DMTs were divided into those targeting amyloid-related targets, tau-related mechanisms, and those with “other” mechanisms (i.e., neuroprotection, anti-inflammation, metabolic effects). The distinction between symptomatic agents and DMTs can be arbitrary and some agents may have both properties. For the purposes of this study, we chose what appears to be the principal MOA.

We retrospectively compare current repurposing trial data with historical data from previous AD pipeline reviews, starting from 2016 onward [10–14]. We used identical methodology to ascertain inclusion of clinical trials as detailed above.

Our study analyzes parallel activity between the US and European AD pipelines using trial information reported on the EUCTR. This public register enables access to data from the European Union Drug Regulating Authorities Clinical Trials (EudraCT) database, which contains protocols and results of studies conducted in the European Union (EU) or the European Economic Area (EEA). We used EUCTR to query Phase I, Phase II, and Phase III AD trials with ongoing status recorded as of February 27, 2020. We distinguish clinical trials exclusive to the EU/EEA from overlap studies running concurrently in the US.

## Results

### Overview

There are 58 total repurposed drugs in 53 clinical trials as reported on ClinicalTrials.gov. We identified 15 agents in 12 trials in Phase III, 33 agents in 32 trials in Phase II, and 10 agents in nine trials in Phase I. Of these trials, six agents were in Phase II/III trials and two agents were in Phase I/II trial. Several trials have more than one agent; some drugs are being studied across multiple trials.

**Fig. 1:**
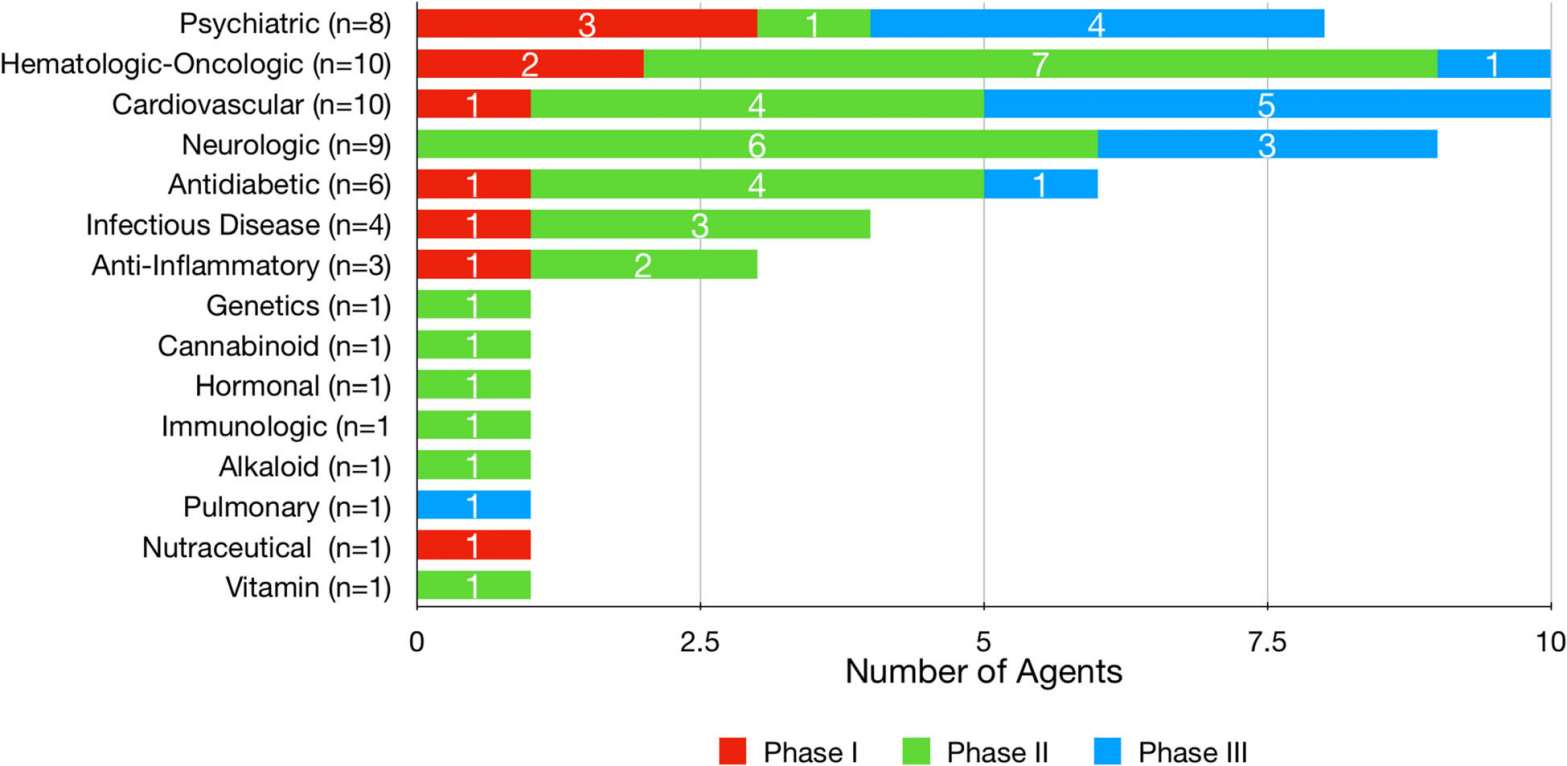
Classification of repurposed agents by their FDA-approved indications (ClinicalTrials.gov as of February 27, 2020)

Analysis of the FDA-approved indications of all repurposed drugs in the pipeline showed that 20% are hematologic-oncologic agents, 18% cardiovascular agents, 14% are psychiatric agents, 12% are antidiabetic treatments, 10% are drugs for other neurologic disorders, and the remaining 26% of drugs are derived from other therapeutic area categories. Repurposed psychiatric drugs in Phase III trials represent 50% of all agents being assessed for the treatment of neuropsychiatric symptoms in AD. Half of all cardiovascular agents under study are in Phase III trials. The majority of the pipeline’s hematologic-oncologic, antidiabetic, and neurologic agents are in Phase II trials (70, 67, and 67%, respectively). Phase II has more repurposed agents than Phase I and Phase III combined. Figure 1 shows the classification of all repurposed drugs by FDA-approved indications within their respective clinical phases.

**Fig. 2:**
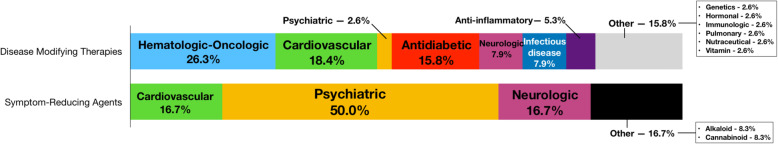
Percentage of repurposed agents currently under study by mechanisms of action (ClinicalTrials.gov as of February 27, 2020)

Review of the AD-related MOAs of all agents shows that 78% are being developed as DMTs, 16% are symptomatic agents addressing neuropsychiatric and behavioral changes, and 7% are cognitive enhancers (Table 1). Escitalopram is being investigated separately as a cognitive enhancer in Phase I and as a neuropsychiatric agent in Phase III. Among DMT trials, the agents are derived primarily from hematologic-oncologic, cardiovascular, and antidiabetic indications (26, 18, and 16%, respectively). In contrast, symptomatic agents are derived mostly from treatments addressing psychiatric illness (50%). Figure 2 shows the repurposed agents’ AD-related MOAs.

**Table 1:** Alzheimer’s disease-related mechanisms of action of repurposed agents in the pipeline (ClinicalTrials.gov as of February 27, 2020)

Approved therapeutic area	Disease-modifying therapies	Cognitive enhancers	Behavioral symptoms	Behavioral + cognitive symptoms	Total # of agents (%)
**Hematologic-oncologic**	10	–	–	–	**10 (20.0)**
**Cardiovascular**	7	1	1	–	**9 (18.0)**
**Psychiatric**	1	1	4	1	**7 (14.0)**
**Antidiabetic**	6	–	–	–	**6 (12.0)**
**Neurologic**	3	–	2	–	**5 (10.0)**
**Infectious disease**	3	–	–	–	**3 (6.0)**
**Anti-inflammatory**	2	–	–	–	**2 (4.0)**
**Genetics**	1	–	–	–	**1 (2.0)**
**Cannabinoid**	–	–	1	–	**1 (2.0)**
**Hormonal**	1	–	–	–	**1 (2.0)**
**Immunologic**	1	–	–	–	**1 (2.0)**
**Alkaloid**	–	1	–	–	**1 (2.0)**
**Pulmonary**	1	–	–	–	**1 (2.0)**
**Nutraceutical**	1	–	–	–	**1 (2.0)**
**Vitamin**	1	–	–	–	**1 (2.0)**
**TOTAL**	**38**	**3**	**8**	**1**	**50 (100.0)**

### Unique repurposed agents

**Fig. 3:**
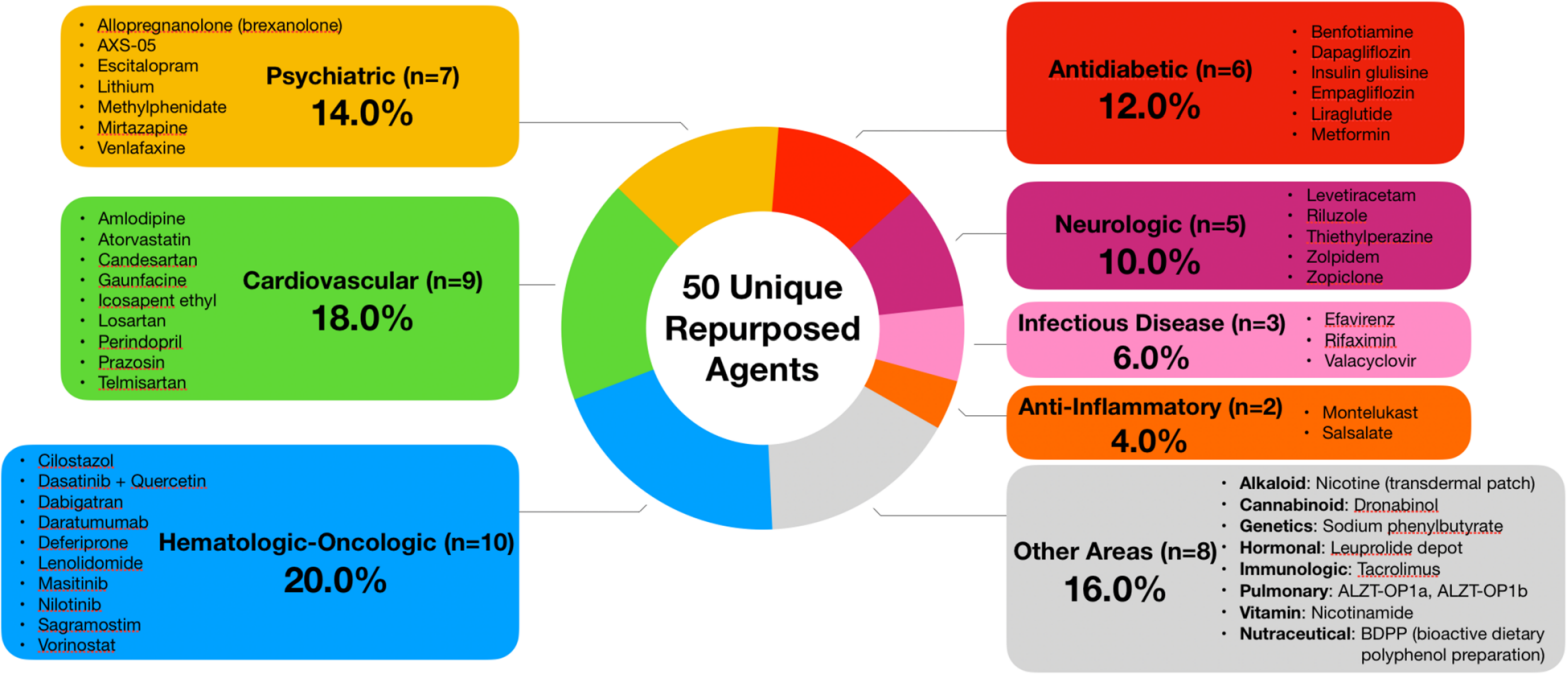
Classification of the therapeutic areas of repurposed agent currently under study (ClinicalTrials.gov as of February 27, 2020)

There are 50 unique repurposed drugs (defined as drugs represented at least once from the 58 total in the pipeline) that are under study among all AD pipeline trials. Twenty percent are hematologic-oncologic agents, 18% are cardiovascular agents, 14% are psychiatric agents, 12% are antidiabetic agents, 10% are neurologic drugs, and the remaining 26% of treatments were used in other categories. Figure 3 provides a graphical summary of the classifications of all unique repurposed agents represented in the current AD pipeline.

The agent most represented among all trials is levetiracetam (four Phase II trials, one Phase III trial). Telmisartan, montelukast, escitalopram, and valacyclovir are each in multiple Phase II and Phase III trials. A propriety low-dose formulation of levetiracetam (AGB101, which is 1/15th of the original dose) is being investigated in Phase II and Phase III trials.

### Phase III

In Phase III trials, there are 15 repurposed agents in 12 trials (Table 2); this represents one-third of all Phase III trials in the AD pipeline [14]. These include eight DMTs, one cognitive-enhancer, and six agents addressing behavioral symptoms. Among DMT agents, one had amyloid-related targets, five had anti-inflammatory MOAs, four were considered metabolic, and two were neuroprotective agents.

**Table 2:** Repurposed agents currently in Phase III in the Alzheimer’s disease development pipeline (as of February 27, 2020)

Agent name	Phase	Agent mechanism class	Therapeutic purpose under study	Drug class	FDA-approved indication(s)	Therapeutic field	ClinicalTrials.gov ID(s) (EudraCT ID)
ALZT-OP1a, ALZT-OP1b (cromolyn with and without ibuprofen)	**III**	Anti-amyloid, anti-inflammatory	Reduce neuronal damage; mast cells may also play a role in amyloid pathology (DMT)	Mast cell stabilizer	Bronchial asthma; mastocytosis	Pulmonary	NCT02547818 (2015-002147-34)
Amlodipine	**II/III**	Anti-inflammatory, metabolic	Vascular risk reduction to preserve cognitive function (DMT)	Calcium channel blocker	Hypertension; coronary artery disease	Cardiovascular	NCT02913664
Atorvastatin	**II/III**	Anti-inflammatory, metabolic	Vascular risk reduction to preserve cognitive function (DMT)	Statin	Adjunct therapy for myocardial infarction, revascularization procedure, angina	Cardiovascular	NCT02913664
AXS-05 (DM + Bupropion)	**II/III**	Neurotransmitter-based	Improve neuropsychiatric symptoms (agitation)	Norepinephrine-dopamine reuptake inhibitor	Major depressive disorder; easonal affective disorder; smoking cessation	Psychiatric	NCT03226522
Escitalopram	**III**	Neurotransmitter-based	Improve neuropsychiatric symptoms (agitation)	Selective serotonin reuptake inhibitor	Major depressive disorder; generalized anxiety disorder	Psychiatric	NCT03108846
Guanfacine	**III**	Neurotransmitter-based	Modulation of noradrenergic deficit (cognitive enhancer)	Alpha-receptor agonist	Attention deficit hyperactivity disorder	Cardiovascular	NCT03116126
Icosapent ethyl	**II/III**	Neuroprotective	Protect neurons from disease pathology (DMT)	Purified w-3 fatty acid (eicosapentaenoic acid)	Hypertriglyceridemia	Cardiovascular	NCT02719327
Levetiracetam (AGB101)	**III**	Neuroprotective	Decrease amyloid-induced neuronal hyperactivity (DMT)	Anticonvulsant	Adjunct therapy for partial-onset seizures; juvenile myoclonic epilepsy; primary generalized tonic-clonic seizures	Neurologic	NCT03486938
Losartan	**II/III**	Anti-inflammatory, metabolic	Vascular risk reduction to preserve cognitive function (DMT)	Angiotensin II receptor blocker	Hypertension; diabetic neuropathy; risk reduction of stroke	Cardiovascular	NCT02913664
Masitinib	**III**	Anti-inflammatory	Activity on mast cells, modulation of inflammatory processes (DMT)	Selective tyrosine kinase inhibitor	Mast cell tumor (veterinary)	Hematologic-oncologic	NCT01872598 (2010-021218-50)
Metformin	**II/III**	Metabolic	Improve insulin sensitivity, may improve cognition (DMT)	Insulin sensitizer (biguanide)	Type 2 diabetes	Antidiabetic	NCT04098666
Methylphenidate	**III**	Neurotransmitter-based	Improve neuropsychiatric symptoms (apathy)	Stimulant	Attention deficit hyperactivity disorder	Psychiatric	NCT02346201
Mirtazapine	**III**	Neurotransmitter-based	Improve neuropsychiatric symptoms (agitation)	Tetracyclic antidepressant	Major depressive disorder	Psychiatric	NCT03031184
Zolpidem	**III**	Neurotransmitter-based	Improve neuropsychiatric symptoms (sleep disorders)	Sedative-Hypnotic	Insomnia	Neurologic	NCT03075241
Zopiclone	**III**	Neurotransmitter-based	Improve neuropsychiatric symptoms (sleep disorders)	Sedative-Hypnotic	Insomnia	Neurologic	NCT03075241

*Abbreviations*: *DMT* disease-modifying therapy, *DM* dextromethorphan

Therapeutic areas represented among the repurposed agents included five cardiovascular drugs, four with psychiatric indications, three neurological drugs, one hematologic-oncologic agent, one antidiabetic agent, and one pulmonary agent. Among these drugs, sedative-hypnotics were the most common.

In Phase III, there were two prevention trials with cognitively normal (preclinical) participants; three trials of patients with prodromal AD/mild cognitive impairment (MCI); five trials of patients with mild to moderate AD; and two trials of patients with mild to moderate/severe AD.

### Phase II

Phase II of the AD pipeline has 32 trials involving 33 repurposed agents (Table 3); this is 44% of all AD trials in Phase II [14]. Of the repurposed agents, there were 27 DMTs, one cognitive-enhancing agent, and three drugs for behavioral symptoms. Among the DMTs, three involve amyloid targets, one addresses tau-related targets, one has a mechanism relevant to both amyloid- and tau-related targets, and 22 have other MOAs (e.g., neuroprotection, metabolic, or anti-inflammatory). Of the symptomatic agents, all four are neurotransmitter-based.

**Table 3:** Repurposed agents currently in Phase II in the Alzheimer’s disease development pipeline (as of February 27, 2020)

Agent name	Phase	Agent mechanism class	Therapeutic purpose under study	Drug class	FDA-approved indication(s)	Therapeutic field	ClinicalTrials.gov ID(s) (EudraCT ID)
Benfotiamine	**II**	Metabolic	Improve multiple cellular processes; minimize decline in glucose utilization (DMT)	Synthetic thiamine	Diabetic nephropathy; type 2 diabetes	Antidiabetic	NCT02292238
Candesartan	**II**	Anti-amyloid, neuroprotective, metabolic	Improve vascular function and reduce amyloid (DMT)	Angiotensin II Receptor Blocker	Hypertension; heart failure	Cardiovascular	NCT02646982
Cilostazol	**II**	Neuroprotective	Reduce accumulation of amyloid and reduce tau phosphorylation; improve cerebral circulation (DMT)	Antiplatelet	Intermittent claudication	Hematologic-oncologic	NCT02491268
Dapagliflozin	**I/II**	Metabolic	Improve insulin sensitivity (DMT)	Sodium-glucose co-transporter 2 inhibitor	Adjunct therapy for type 2 diabetes	Antidiabetic	NCT03801642
Daratumumab	**II**	Anti-inflammatory	Broad range immunomodulatory effects; regulate microglia (DMT)	Human antibody targeting CD38	Multiple myeloma	Hematologic-oncologic	NCT04070378
Dasatinib + Quercetin (Combo)	**I/II**	Anti-inflammatory	Senolytic therapy approach to reduce senescent cells and tau aggregation (DMT)	Tyrosine kinase inhibitor + antioxidant/anti-inflammatory (Flavonoid)	Philadelphia chromosome-positive chronic myeloid leukemia in chronic phase	Hematologic-oncologic	NCT04063124
Deferiprone	**II**	Anti-Amyloid, neuroprotective	Reduce reactive oxygen species that damage neurons; effect on amyloid and BACE pathology (DMT)	Iron chelator	Iron overload (thalassemia syndromes)	Hematologic-oncologic	NCT03234686
Dronabinol	**II**	Neurotransmitter-based	Improve neuropsychiatric symptoms (agitation)	Cannabinoid	Anorexia (associated with AIDS); nausea/vomiting (associated with chemotherapy)	Cannabinoid	NCT02792257
Insulin glulisine	**II**	Metabolic	Enhance cell signaling and growth; promote neuronal metabolism (DMT)	Insulin	Diabetes	Antidiabetic	NCT02503501
Lenolidomide	**II**	Anti-inflammatory	Reduce inflammatory cytokines (TNF-a, IL-6, IL-8); reduce inflammatory and AD-associated biomarkers; improve cognition (DMT)	Anti-neoplastic	Multiple myeloma	Hematologic-oncologic	NCT04032626
Levetiracetam	**II**	Neuroprotective	Reduce amyloid induced neuronal hyperactivity (DMT)	Anticonvulsant	Adjunct therapy for partial-onset seizures; juvenile myoclonic epilepsy; primary generalized tonic-clonic seizures	Neurologic	NCT03489044 (2016–003109-32)
Levetiracetam	**II**	Neuroprotective	Reduce amyloid induced neuronal hyperactivity (DMT)	Anticonvulsant	Adjunct therapy for partial-onset seizures; juvenile myoclonic epilepsy; primary generalized tonic-clonic seizures	Neurologic	NCT02002819
Levetiracetam	**II**	Neuroprotective	Reduce amyloid induced neuronal hyperactivity (DMT)	Anticonvulsant	Adjunct therapy for partial-onset seizures; juvenile myoclonic epilepsy; primary generalized tonic-clonic seizures	Neurologic	NCT03875638
Levetiracetam (AGB101)	**II**	Neuroprotective	Reduce amyloid induced neuronal hyperactivity (DMT)	Anticonvulsant	Adjunct therapy for partial-onset seizures; juvenile myoclonic epilepsy; primary generalized tonic-clonic seizures	Neurologic	NCT03461861
Liraglutide	**II**	Metabolic	Enhance cell signaling; improve CNS glucose metabolism (DMT)	Glucagon-like peptide-1 agonist	Adjunct therapy for type 2 diabetes; risk reduction of major cardiovascular events	Antidiabetic	NCT01843075 (2013-000962-13)
Lithium	**II**	Neurotransmitter-based	Improve neuropsychiatric symptoms (agitation/aggression with or without psychosis)	Antimanic	Bipolar disorder	Psychiatric	NCT02129348
Leuprolide depot	**II**	Metabolic	Alleviates negative effects of elevated GnRH and gonadtropins on the brain (DMT)	Gonadotropin-releasing hormone agonist	Endometriosis; uterine leiomyomata	Hormonal	NCT03649724
Montelukast (buccal film)	**II**	Anti-inflammatory	Reduce inflammatory pathways (neuronal injury, blood-brain barrier integrity, amyloid-ß accumulation); effect on cognition and AD biomarkers (DMT)	Leukotriene receptor antagonist	Asthma; exercise-induced bronchoconstriction; allergic rhinitis	Anti-inflammatory	NCT03402503
Montelukast (tablet)	**II**	Anti-inflammatory	Reduce inflammatory pathways that mediate neuronal injury, blood-brain barrier integrity, amyloid-ß accumulation (DMT)	Leukotriene receptor antagonist	Asthma; exercise-induced bronchoconstriction; allergic rhinitis	Anti-inflammatory	NCT03991988
Nicotinamide	**II**	Anti-tau, neuroprotective	Reduce tau-induced microtubule depolymerization; reduce phosphorylation of tau (DMT)	Vitamin B3	Pellagra	Vitamin	NCT03061474
Nicotine (transdermal patch)	**II**	Neurotransmitter-based	Enhance acetylcholine signaling (cognitive enhancer)	Alkaloid	Smoking cessation withdrawal symptoms	Alkaloid	NCT02720445
Nilotinib	**II**	Anti-amyloid, anti-tau	Autophagy enhancer; promotes clearance of amyloid and tau proteins (DMT)	Tyrosine kinase inhibitor	Philadelphia chromosome-positive chronic myeloid leukemia in chronic phase	Hematologic-oncologic	NCT02947893
Perindopril	**II**	Neuroprotective, anti-inflammatory	Improves vascular functioning (DMT)	Angiotensin-converting-enzyme inhibitor	Essential hypertension; stable coronary artery disease	Cardiovascular	NCT02085265
Prazosin	**II**	Neurotransmitter-based	Improve neuropsychiatric symptoms (agitation)	Alpha-receptor antagonist	Hypertension	Cardiovascular	NCT03710642
Rifaximin	**II**	Anti-inflammatory	Improve cognition and function by lowering blood ammonia; lower circulatory pro-inflammatory cytokines secreted by harmful gut bacteria (DMT)	Non-systemic antibiotic	Traveler’s diarrhea; overt hepatic encephalopathy; irritable bowel syndrome (diarrhea subtype)	Infectious disease	NCT03856359
Riluzole	**II**	Neuroprotective	Reduce glutamate-mediated excitotoxicity (DMT)	Benzothiazole	Amyotrophic lateral sclerosis	Neurologic	NCT01703117
Sagramostim	**II**	Anti-amyloid, neuroprotective	Stimulates innate immune system to remove amyloid pathology; increase neuronal connectivity (DMT)	Human granulocyte-macrophage colony-stimulating factor	Bone marrow stimulation	Hematologic-oncologic	NCT01409915
Sodium phenylbutyrate	**II**	Neuroprotective	Blocks nerve cell death and modulates neuroinflammation (DMT)	Anti-hyperammonemia	Adjunct for chronic urea cycle disorders	Genetics	NCT03533257
Tacrolimus	**II**	Neuroprotective	Prevents amyloid-ß dendritic spine loss; normalizes cranial nerve activity in the brain (DMT)	Calcineurin inhibitor	Prevent rejection of organ transplants	Immunologic	NCT04263519
Telmisartan	**II**	Neuroprotective, anti-inflammatory	Improves vascular functioning (DMT)	Angiotensin II Receptor Blocker	Hypertension; diabetic neuropathy; risk reduction of stroke	Cardiovascular	NCT02085265
Thiethylperazine	**II**	Anti-amyloid	Activates transport protein ABCC1 and enhances transport amyloid-ß peptides from the brain into the blood (DMT)	Phenothiazine	Nausea/vomiting	Neurologic	NCT03417986 (2014-000870-20)
Valacyclovir	**II**	Neuroprotective, anti-inflammatory	Reduce amyloid-ß aggregation by preventing overproduction of amyloid in response to infection (DMT)	Antiviral (nucleoside analog)	Herpes labialis; genital herpes	Infectious disease	NCT03282916
Valacyclovir	**II**	Neuroprotective, anti-inflammatory	Reduce amyloid-ß aggregation by preventing overproduction of amyloid in response to infection (DMT)	Antiviral (nucleoside analog)	Herpes labialis; genital herpes	Infectious disease	NCT02997982

*Abbreviation*: *DMT* disease-modifying therapy

Therapeutic areas of Phase II repurposed agents comprise hematologic-oncologic agents (seven), neurologic drugs (six), cardiovascular drugs (four), antidiabetic agents (four), and other classes (e.g., cannabinoid, alkaloid; 12 total). Anticonvulsants were the most represented drug class in Phase II.

There is one prevention trial; 19 trials of patients with prodromal/MCI or prodromal/mild-AD; 11 trials in mild-moderate AD; and one trial of patients with mild-moderate/severe AD. Among DMT trials, there is one prevention trial with cognitively normal participants, 17 for prodromal/mild AD, and eight for participants with mild-moderate AD.

### Phase I

There are ten repurposed agents among nine Phase I trials (Table 4), which is 33% of Phase I trials in the pipeline [14]. Of these, there are eight DMTs and two cognitive enhancers. No agents addressing neuropsychiatric symptoms are represented in the Phase I pipeline. Of the DMTs in Phase I, one agent is directed at amyloid-related targets while seven have other MOAs (e.g., neuroprotection, metabolic, or anti-inflammatory). No tau-related repurposed agents are under investigation in Phase I of the current pipeline.

**Table 4:** Repurposed agents currently in Phase I in the Alzheimer’s disease development pipeline (as of February 27, 2020)

Agent name	Phase	Agent mechanism class	Therapeutic purpose under study	Drug class	FDA-approved indication(s)	Therapeutic field	ClinicalTrials.gov ID(s)
Allopregnanolone (Brexanolone)	**I**	Neuroprotective, metabolic	Enhanced neurogenesis slow hippocampal atrophy; improve synaptic connectivity (DMT)	GABA-A receptor modulator; neuroactive steroid (antidepressant)	Postpartum depression	Psychiatric	NCT03748303
BDPP (Bioactive Dietary Polyphenol Preparation)	**I**	Neuroprotective	Prevents amyloid and tau aggregation (DMT)	Polyphenol	Dietary supplement	Nutraceutical	NCT02502253
Dabigatran	**I**	Neuroprotective	Reduce neurovascular damage (DMT)	Anticoagulant	Reduce risk of stroke; embolism associated with atrial fibrillation, deep venous thrombosis, pulmonary embolism	Hematologic-oncologic	NCT03752294
Efavirenz	**I**	Anti-amyloid	Increase cholesterol removal and enhance amyloid reduction (DMT)	Non-nucleoside reverse-transcriptase inhibitor	HIV/AIDS	Infectious disease	NCT03706885
Escitalopram	**I**	Neurotransmitter-based	Increase neurotransmission (cognitive enhancer)	Selective serotonin reuptake inhibitor	Major depressive disorder; generalized anxiety disorder	Psychiatric	NCT03274817
Empagliflozin	**I**	Metabolic	Increase ketone levels in the brain; improve neuronal health and delay onset and progression of cognitive impairment (DMT)	Sodium-glucose co-transporter 2 inhibitor	Adjunct therapy for type 2 diabetes	Antidiabetic	NCT03852901
Salsalate	**I**	Anti-inflammatory	Reduce neuronal injury (DMT)	Non-steroidal anti-inflammatory drug	Rheumatoid arthritis; osteoarthritis	Anti-inflammatory	NCT03277573
Telmisartan	**I**	Neuroprotective, anti-inflammatory	Improve vascular functioning and effects on amyloid pathology (DMT)	Angiotensin II Receptor Blocker	Hypertension; diabetic neuropathy; risk reduction of stroke	Cardiovascular	NCT02471833
Venlafaxine	**I**	Neurotransmitter-based	Increase neurotransmission (cognitive enhancer)	Serotonin-norepinephrine reuptake inhibitor	Major depressive disorder; general anxiety disorder; seasonal affective disorder; panic disorder	Psychiatric	NCT03274817
Vorinostat	**I**	Neuroprotective	Multiple cellular processes including tau aggregation and amyloid deposition (DMT)	Histone deacetylase inhibitor (anticancer)	Cutaneous T-cell lymphoma	Hematologic-oncologic	NCT03056495

Abbreviations: DMT, disease modifying therapy

In Phase I, repurposed agents include three psychiatric drugs, two hematologic-oncologic agents, one cardiovascular drug, one antidiabetic agent, and three agents in other drug classes (e.g., anti-inflammatory drug, infectious disease agent).

### Trial sponsors

Across all trials of repurposed agents, 9% are sponsored by the biopharma industry, 79% by Academic Medical Centers (with funding from the National Institutes of Health, industry, and/or other entities such as a consortium or a philanthropic foundation), and 11% by others. Table 5 shows sponsors for each phase of development.

**Table 5:** Trial sponsor for repurposed agents by phase of drug development (as of February 27, 2020)

Sponsor	Phase I	Phase II	Phase III	Total (%)
**Academic Medical Centers**	6	14	4	**24 (45.3)**
**Biopharma**	0	2	3	**5 (9.4)**
**NIH**	1	0	0	**1 (1.9)**
**NIH and Academic Medical Centers**	0	4	2	**6 (11.3)**
**NIH and consortium**	0	1	0	**1 (1.9)**
**NIH and Industry**	0	0	1	**1 (1.9)**
**Academic Medical Centers and Industry**	0	2	0	**2 (3.8)**
**Academic Medical Centers and Foundation**	1	4	0	**5 (9.4)**
**Academic Medical Centers, Industry, and NIH**	0	0	1	**1 (1.9)**
**Other combinations**	1	4	0	**5 (9.4)**
**Other federal sources**	0	1	1	**2 (3.8)**
**Total**	**9**	**32**	**12**	**53 (100.0)**

*Abbreviations*: *NIH* National Institutes of Health

### Repurposed agents in the pipeline from 2016 to 2020

Table 6 summarizes the number of repurposing trials in previous annual AD pipeline reports [10–14]. Since 2016, the number of trials involving repurposed agents has increased by 89%. The number of trials has grown each year, with the greatest rate of increase occurring between 2019 and 2020 (23% growth). Between 2016 and 2020, repurposed DMT trials have increased 180%; repurposed trials focused on symptom-reduction have decreased by 67%.

**Table 6:** Repurposing agent trials between 2016 and 2020

	2016		2017		2018		2019		2020	
	DMTs	Symptomatic agents	DMTs	Symptomatic agents	DMTs	Symptomatic agents	DMTs	Symptomatic agents	DMTs	Symptomatic agents
**Phase I**	1	3	1	2	3	2	7	1	8	1
**Phase II**	10	7	11	9	15	10	19	7	28	4
**Phase III**	4	3	4	4	2	5	3	6	6	6
**Total**	**15**	**13**	**16**	**15**	**20**	**17**	**29**	**14**	**42**	**11**

*Abbreviations*: *DMT* disease-modifying therapy

**Fig. 4:**
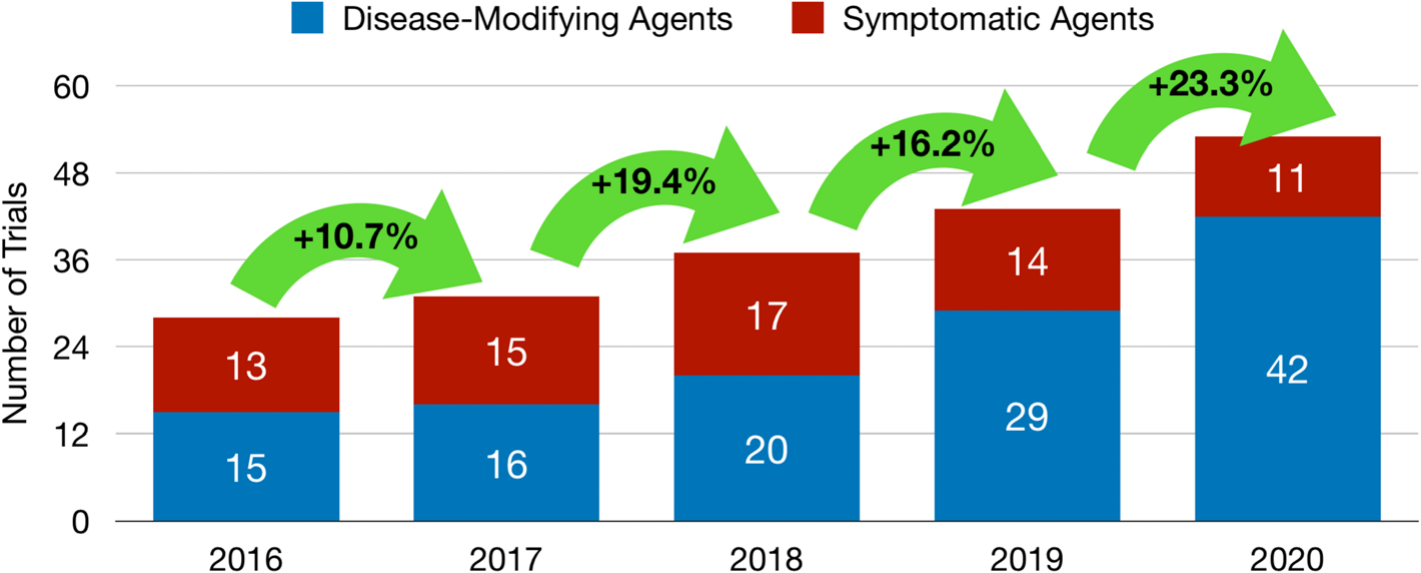
Repurposing trials over time in the Alzheimer’s disease drug development pipeline (2016–2020)

Phase I trials of repurposed agents have increased 125% since 2016; Phase II and Phase III trials have risen 88% and 71%, respectively. DMT trials of repurposed agents currently make up 79% of all repurposed agents in the current pipeline (up from 54% in 2016; Fig. 4).

### Repurposed agents in the European AD pipeline

Out of 138 AD trials with an ongoing status registered with the EUCTR, we identified 14 trials involving 14 FDA-approved drugs (Table 7). Eleven of these were dual-registered with the US and EU/EEA databases. Five trials were in Phase III and nine were in Phase II. Six had a completed or terminated status when compared to the information listed on ClinicalTrials.gov. Three trials were registered exclusively with the EUCTR: epoetin alfa, levodopa-carbidopa, and cannabidiol.

**Table 7:** Repurposing agents currently in the European Alzheimer’s Disease development pipeline (as of February 27, 2020)

Agent name	Phase(s)	Agent mechanism class	Therapeutic purpose under study	Drug class	FDA-approved indication(s)	Therapeutic field	EudraCT ID (Clinicaltrials.gov ID)
ALZT-OP1a, ALZT-OP1b (cromolyn with and without ibuprofen)	**III**	Anti-amyloid, anti-inflammatory	Reduce neuronal damage; mast cells may also play a role in amyloid pathology (DMT)	Mast cell stabilizer	Bronchial asthma; mastocytosis	Pulmonary	2015-002147-34 (NCT02547818)
Cannabidiol	**II**	Neurotransmitter-based	Improve neuropsychiatric symptoms (agitation)	Cannabinoid	Seizures (Lennox-Gastaut syndrome or Dravet syndrome)	Neurologic	2019-002106-52
Dronabinol	**II**	Neurotransmitter-based	Improve neuropsychiatric symptoms (agitation)	Synthetic delta-9-THC	AIDS-associated anorexia; antiemetic for chemotherapy-induced nausea and vomiting	Neurologic	2011-005289-39* (NCT01608217) 2010-024577-39* (NCT01302340)
Epoetin alfa	**II**	Anti-inflammatory, metabolic	Reduce neuronal injury (DMT); improve neuropsychiatric symptoms (depression)	Erythropoiesis-stimulating agent	Anemia (chronic kidney disease, zidovudine treatment, chemotherapy); reduction of red blood cell transfusion	Hematologic-oncologic	2008-000453-35
Levetiracetam	**II**	Neuroprotective	Reduce amyloid induced neuronal hyperactivity (DMT)	Anticonvulsant	Adjunct therapy for partial-onset seizures; juvenile myoclonic epilepsy; primary generalized tonic-clonic seizures	Neurologic	2016-003109-32 (NCT03489044)
Levodopa-Carbidopa	**II**	Neurotransmitter-based	Enhance dopamine neurotransmission (cognitive enhancer)	Dopamine prodrug	Parkinson’s disease	Neurologic	2009-011093-15
Liraglutide	**II**	Metabolic	Enhance cell signaling; improve CNS glucose metabolism (DMT)	Glucagon-like peptide-1 agonist	Adjunct therapy for type 2 diabetes; risk reduction of major cardiovascular events	Antidiabetic	2013-000962-13 (NCT01843075)
Masitinib	**III**	Anti-inflammatory	Activity on mast cells, modulation of inflammatory processes (DMT)	Selective tyrosine kinase inhibitor	Mast cell tumor (veterinary)	Hematologic-oncologic	2010-021218-50 (NCT01872598)
Rosiglitazone	**III**	Metabolic	PPAR-gamma agonist (cognitive enhancer)	Thiazolidinedione	Adjunct therapy for type 2 diabetes	Antidiabetic	2012-002764-27** (NCT00550420) 2006-001403-11* (NCT00348309) 2006-001402-92* (NCT00348140)
Rotigotine	**II**	Neurotransmitter-based	Enhance dopamine neurotransmission (cognitive enhancer)	Non-ergoline dopamine agonist	Parkinson’s disease; restless leg syndrome	Neurologic	2015-002965-43* (NCT03250741)
Thiethylperazine	**II**	Anti-amyloid	Activates transport protein ABCC1 and enhances transport amyloid-ß peptides from the brain into the blood (DMT)	Phenothiazine	Nausea/vomiting	Neurologic	2014-000870-20 (NCT03417986)

*Abbreviations*: *DMT* disease-modifying therapy, *THC* tetrahydrocannabinol

*Trials that have a “Completed” status as reported on CiinicalTrials.gov

**Trials that have a “Terminated” status as reported on ClinicalTrials.gov

There were six DMTs, five cognitive-enhancing agents, and three drugs addressing neuropsychiatric symptoms among the repurposed agents. Therapeutic areas represented included four neurologic drugs, two hematologic-oncologic agents, one drug with pulmonary indications, and one antidiabetic agent.

## Discussion

Drug repurposing provides the unique opportunity to significantly shorten the development period of a therapeutic candidate for AD. These agents have been extensively assessed for pharmacokinetic properties and toxicities normally required in nonclinical and Phase 1 stages of development. Over half of all agents fail in development for these two reasons, and repurposing circumvents these challenges [18]. FDA-approved drugs have already undergone human trials, and safety, efficacy, and adverse event profiles are known. The results of previous trials may suffice to bypass nonclinical stages and earlier clinical phases of study; this has the advantage of foregoing the financial and time-costs associated with a drug development program. Total costs for developing a new agent may be as high as $5.7 billion with $1.66 billion required for non-clinical studies and $1.19 billion for Phase I [19]. Total development costs may be reduced by 50% compared to the cost of developing a new chemical entity.

FDA-approved agents have been optimized for the original indication. Different doses might be needed, and different adverse event profiles may occur in an AD population compared to other populations for which the agent was originally developed. Some steps in the development process might be repeated to account for these contingencies. Repurposed agents are often generic and have limitations on intellectual property rights that compromise attracting investments that could propel their development [20].

Examination of the repurposed agents in the current AD pipeline revealed that DMT trials were chiefly based on agents derived from hematologic-oncologic (tyrosine kinase inhibitors), cardiovascular (angiotensin II receptor antagonists, angiotensin-converting enzyme inhibitors), and neurologic indications (anticonvulsants), as well as diabetes. Growing evidence supports the association between the key pathologic changes these drugs target and the underlying biology of AD [21–24]. The links between dementia-related symptoms and the abnormal activation of tyrosine kinase pathways, cardiovascular disease, and impaired insulin sensitivity are reflected by the presence of these agents in the pipeline of repurposed drugs. With increasing knowledge of these processes in AD, repurposing may comprise a precision medicine approach to AD therapeutics [25]. A preponderance of psychiatric compounds comprise the symptomatic agent class, and the vast majority (including some non-antipsychotics) are aimed at alleviating AD-associated agitation. This may show a priority in the field to identify treatments for a distressing aspect of AD for patients and caregivers. These trials were facilitated by the development of new diagnostic criteria for agitation that assist in patient identification and outcome determination [26].

Certain repurposed candidates may offer therapeutic benefits without directly engaging AD targets. Reduced cerebral blood flow correlates with AD pathology [27]. A pilot study has suggested that the antiplatelet agent, cilostazol (NCT02491268), may slow cognitive decline through pleiotropic effects, including regional enhancement of cerebral perfusion [28–30]. Some drugs target the role of neuroinflammation in AD: inflammation affecting the function of the blood-brain barrier is a component of interest in two trials of montelukast (NCT03402503, NCT03991988, [31, 32]). Trialists studying rifaximin (NCT03856359) posit an improvement in cognitive function by reduction in pro-inflammatory cytokines and ammonia levels [32].

Repurposing agent trials make up 39% of the total AD pipeline in 2020 (compared to 24% in 2016). Phase II has the largest number of trials—in both repurposing trials and in the overall pipeline (60 and 55%, respectively). This likely results from entry of repurposed agents directly into Phase II or Phase I/II. DMT trials comprise approximately 79% of the 2020 AD pipeline, compared to 75% when looking at the subset of repurposed agents. Although amyloid is the most common specific target in Phase II and Phase III DMT trials overall, the majority of repurposed DMTs involve tau targets.

Forty-six percent of all AD trials are funded at least in part by biopharmaceutical industry sources. In contrast, the majority of repurposed trials (79%) receive partial or complete funding from Academic Medical Centers and only 9% are sponsored by the biopharmaceutical industry. This reflects the challenges of obtaining intellectual property protection for repurposed agents that discourage for-profit enterprises from exercising this development pathway. The observation emphasizes the importance of Academic Medical Centers and federal and philanthropic funding in the AD drug development ecosystem.

A variety of strategies are being assessed to try to secure intellectual property positions for repurposed agents. Using different doses relative to an agent’s original indication is one approach and depends on the drug exposure required for each indication. This strategy is exemplified by the HOPE4MCI study (NCT03486938), which is using one-tenth of the dose of levetiracetam used for anticonvulsant purposes. Another approach is the use of novel formulations of an agent. Insulin glulisine (NCT02503501) is a rapid-acting version of the insulin; a long-acting form (NCT01595646) was developed for trials in AD [33]. Montelukast is being investigated as a cognitive enhancer in two separate trials (NCT03991988, NCT03402503) using an oral tablet and a buccal film patch, respectively. This highlights the opportunity to develop novel delivery mechanisms for repurposed agents as a means of enhancing efficacy and securing intellectual property rights.

The marked increase in repurposing may reflect the recent efforts aimed at elucidating the benefits of repurposing as a strategy in AD drug development [7, 34]. Funding from federal, advocacy, and philanthropic sources disproportionately support trials of repurposed agents and this may have driven the increase in repurposing. Repurposing may have been accelerated by advances in methodologies that are being used to identify AD-efficacious pharmaceuticals that include phenotypic or high content screening, in silico techniques, bioinformatic or computational strategies, and medical genetic-based approaches [9, 35–39]. Electronic health records may provide evidence of beneficial effects on AD or other neurodegenerative disorders by therapies approved for other indications and represent another means for discovering repurposing agents [39, 40]. The effects of long-term exposure to these agents, however, may not be recapitulated in the 18–24 months of most current AD trials.

Between 2016 and 2020, 20 trials of repurposed agents reached a “completed” status while four were “terminated” for various reasons. Post hoc analysis of a pilot Phase II Metformin study (NCT01965756) found the agent to be safe and well-tolerated, with improvements in executive function [41]. In another study, a short course of NewGam 10% IVIG (intravenous immunoglobulin; NCT01300728) given during the mild cognitive impairment (MCI) stage of AD decreased brain atrophy and ameliorated cognitive decline, but beneficial effects did not persist after 2 years [42]. A phase I study (NCT01780519) found that lorazepam may unmask pre-symptomatic cognitive dysfunction in individuals with underlying genetic risk for AD [43]. Primary outcome analyses of the NILVAD study (NCT02017340) failed to show any treatment benefit with nilvadipine when compared to the matched placebo population, though the drug was found to be safe and well-tolerated [44]. The AMBAR study by Grifols (NCT01561053) offers a novel approach to potential AD treatment via therapeutic albumin replacement with or without immunoglobulin [45]. Outcome analyses revealed a statistically significant 61% decrease in progression of moderate AD who received treatment. None of the trial “failures” results from problems associated with safety or tolerability, which may be indicative of the extensive studies repurposed agents have already undergone.

A single repurposed compound may target different outcomes; escitalopram is being studied simultaneously in trials for agitation and for improvements in cognition (NCT03108846, NCT03274817). Combinations of FDA-approved drugs represent another approach to repurposing; ALZT-OP1 (NCT02482324) uses a two-part combination of repurposed agents—a mast-cell stabilizer (cromolyn) and the non-steroidal anti-inflammatory agent ibuprofen. A combination of losartan, amlodipine, and atorvastatin is a combination trial (NCT02913664) involving repurposed agents to address the vascular dimensions of AD.

We assessed repurposing in the EUCTR and compared it to the analyses of ClinicalTrials.gov. Using the same criteria used to query the US governmental database, we found that repurposing trials make up approximately 10% of all ongoing EU/EEA AD trials (in contrast to 39% in the US AD pipeline). Similar to the US pipeline, the majority of trials (64%) are in Phase II and involve DMT agents (43%). The majority of agents identified in the EU/EEA trials were among the agents identified in ClinicalTrials.gov; only three trials were unique to the EUCTR.

Some major discrepancies were encountered when comparing both trial registers. Six of the 14 EUCTR trials, or 43%, were found to have a “completed” or “terminated” status on ClinicalTrials.gov (Table 7). Inaccurate reporting of dual-registered trials on EUCTR is a prevalent issue [46], which may in part reflect the register’s reliance on sponsors to manually enter study results onto the EudraCT database. Suboptimal completion of RCT protocols and results may lead to overestimates of trial activity [47–50].

Repurposing is to be distinguished from developing extended indications for a proprietary agent as part of life-cycle management. Pimavanserin, for example, is approved for the treatment of hallucinations and delusions in Parkinson’s disease psychosis; an extended application for dementia-related psychosis is currently being developed for several dementia syndromes [51, 52]. Likewise, suvorexant is approved for insomnia and completed a successful trial for insomnia in AD and the labeling of the compound has been adjusted to include this information. Brexpiprazole is approved for the treatment of schizophrenia and as adjunctive therapy for major depressive disorder; it is being assessed in the AD pipeline as a therapy for agitation [53]. These programs represent extensions of indications and not repurposing.

Repurposing can be distinguished from repositioning in which a non-approved agent in a sponsor’s development program changes from one indication to another because disease-related, compound-related, or commercial circumstances suggest that success in further development may be more likely in one condition than another [54, 55].

### Limitations

There are limitations of the current analyses. Because not all Phase I trial activity may be recorded in the clinicaltrials.gov database, our data may underestimate the actual number of trials in this phase. Estimates of drugs designated as DMT versus symptomatic agents may be equivocal depending on what is perceived as an agent’s principle MOA. We stopped entering new data into our database at a time that allowed submission, peer review, and publication (final data collection stopped on February 27, 2020) and there may be newer trials or changes to current trials not reflected in this document.

## Conclusions

In summary, repurposing is a promising avenue for AD drug development, comprises a substantial part of the current AD pipeline, and is increasing in absolute numbers of agents and as a proportion of the pipeline. Repurposing may reduce drug development costs by up to 50% and accelerate times lines for progression in the pipeline. Repurposing draws on development programs from numerous other indications. Repurposing is an important part of the current AD drug development ecosystem.

## Data Availability

The datasets used and/or analyzed during the current study are available from the corresponding author on reasonable request.

## Abbreviations

AD: Alzheimer’s disease
AMBAR: Alzheimer’s Management by Albumin Replacement
DMT: Disease-modifying therapy
FDA: Food and Drug Administration
IVIG: Intravenous immunoglobulin
MCI: Mild cognitive impairment
MOA: Mechanism of action
NILVAD: Nilvadipine to Treat Alzheimer’s Disease
