# Correction: Repurposed agents in the Alzheimer’s disease drug development pipeline

**DOI:** 10.1186/s13195-023-01284-9

**Published:** 2023-08-21

**Authors:** Justin Bauzon, Garam Lee, Jeffrey Cummings

**Affiliations:** 1 School of Medicine, University of Nevada, Las Vegas (UNLV), Las Vegas, NV 89154 USA; 2 Cleveland Clinic Lou Ruvo Center for Brain Health, Las Vegas, NV 89106 USA; 3 Chambers-Grundy Center for Transformative Neuroscience, Department of Brain Health, School of Integrated Health Sciences, University of Nevada, Las Vegas (UNLV), Las Vegas, NV 89154 USA

**Correction** **: Alzheimers Res Ther 12, 98 (2020)**

**https://doi.org/10.1186/s13195-020-00662-x**

Following the publication of the original article [1], the authors would like to correct the three occurrences of Dr. Jeffrey Cummings’s initial in Competing interests section from "JL" to "JC”.

The original article [1] has been updated.
